# Complete Genome Sequence of a Precore-Defective Hepatitis B Virus Genotype D2 Strain Isolated in Bangladesh

**DOI:** 10.1128/MRA.00083-20

**Published:** 2020-03-12

**Authors:** Md Golzar Hossain, Md Muket Mahmud, Md Arifur Rahman, Sharmin Akter, K. H. M. Nazmul Hussain Nazir, Sukumar Saha, Masami Wada, Eriko Ohsaki, Tomoyuki Honda, Keiji Ueda

**Affiliations:** a Division of Virology, Department of Microbiology and Immunology, Graduate School of Medicine, Osaka University, Osaka, Japan; b Department of Microbiology and Hygiene, Bangladesh Agricultural University, Mymensingh, Bangladesh; c Department of Physiology, Bangladesh Agricultural University, Mymensingh, Bangladesh; Portland State University

## Abstract

Hepatitis B virus (HBV) genomic mutations affect viral replication, disease progression, and diagnostic and vaccination efficiency. There is limited information regarding characterization and mutational analysis of HBV isolated in Bangladesh. Here, we report the complete nucleotide sequence of a precore-defective HBV genotype D2 strain isolated in Bangladesh.

## ANNOUNCEMENT

Hepatitis B virus (HBV), a causative agent for acute/chronic hepatitis and hepatocellular carcinoma (HCC), has a circular partially double-stranded DNA genome, is about 3.2 kb long, and belongs to the *Hepadnaviridae* family and *Orthohepadnavirus* genus (1, 2). Nucleotide sequence variation in the genome has been used to classify HBV into different genotypes (A to J) (3, 4). However, the prevalences of HBV genotypes and subtypes vary depending on the geographical region, and genomic variations have been associated with HBV pathogenesis (2, 5). The HBV genome encodes four major open reading frames (ORFs), namely, polymerase, HBsAg, core, and X; precore (pre-C)-defective mutations occur due to the introduction of stop codons in this region (6). Pre-C-defective HBV mutants are infectious but are less sensitive to interferon treatment (6). HBV is highly prevalent in Bangladesh, with the genotypes A, C, and D being responsible for 66% of HCC in this country (7). We report the full-genome sequence of a pre-C-defective HBV mutant isolated in Bangladesh from a clinically infected patient and report a mutation analysis.

HBV was detected in a patient serum sample (3). Viral DNA from serum samples was extracted using a QIAamp DNA minikit according to the manufacturer’s protocol. Amplification of the HBV complete overlapping ORFs P, pre-C, and X and the partial S ORF was carried out using specific primers (Table 1), as previously described (3). The PCR products were purified using a MonoFas DNA purification kit (GL Sciences, Inc., Tokyo, Japan) and were used as the templates for sequencing. The sequencing reactions were performed according to our previously reported protocol using the BigDye Terminator v3.1 cycle kit and the BigDye Terminator v1.1 and v3.1 5× sequencing buffer (Applied Biosystems) and were sequenced using an Applied Biosystems 3730 DNA analyzer (California, CA, USA) (3). Raw sequence data were edited, annotated, and analyzed using CLC Sequence Viewer. Determination of the genotype and subgenotype as well as the mutation analysis were performed using Geno2pheno:hbv (https://hbv.geno2pheno.org/) and HIV-Grade:HBV-Tool (https://www.hiv-grade.de/cms/grade/explanations/hbv-tool/) with default parameters. The newly obtained HBV (strain HBV_BAU1) genome sequence was aligned with different genotypes and subgenotypes for the construction of phylogenetic trees using CLC Sequence Viewer (8).

**TABLE 1:** Primers used in this study

Primer name	Sequence (5′–3′)
EcoRI-Pol-1	GTGGAATTCGGATGCCCCTATCTTATCAACAC
Pol-stop-SalI	CACGTCGACTCACGGTGGTCTCCATGCGAC
HBs F2	CTTCATCCTGCTGCTATGCCT
HBsR2	AAAGCCCAGGATGATGGGAT
HBs-stop-SalI	CACGTCGACTTAAATGTATACCCAAAGAC
EcoRI-PreC-1	GTGGAATTCGGATGCAACTTTTTCACCTCTGC
Core-stop-SalI	CACGTCGACTAACATTGAGATTCCCGAG
EcoRI-HBx-1	GTGGAATTCGGATGGCTGCTAGGGTGTGCTG
HBx-stop-SalI	CACGTCGACTTAGGCAGAGGTGAAAAAGTTG

**FIG 1:**
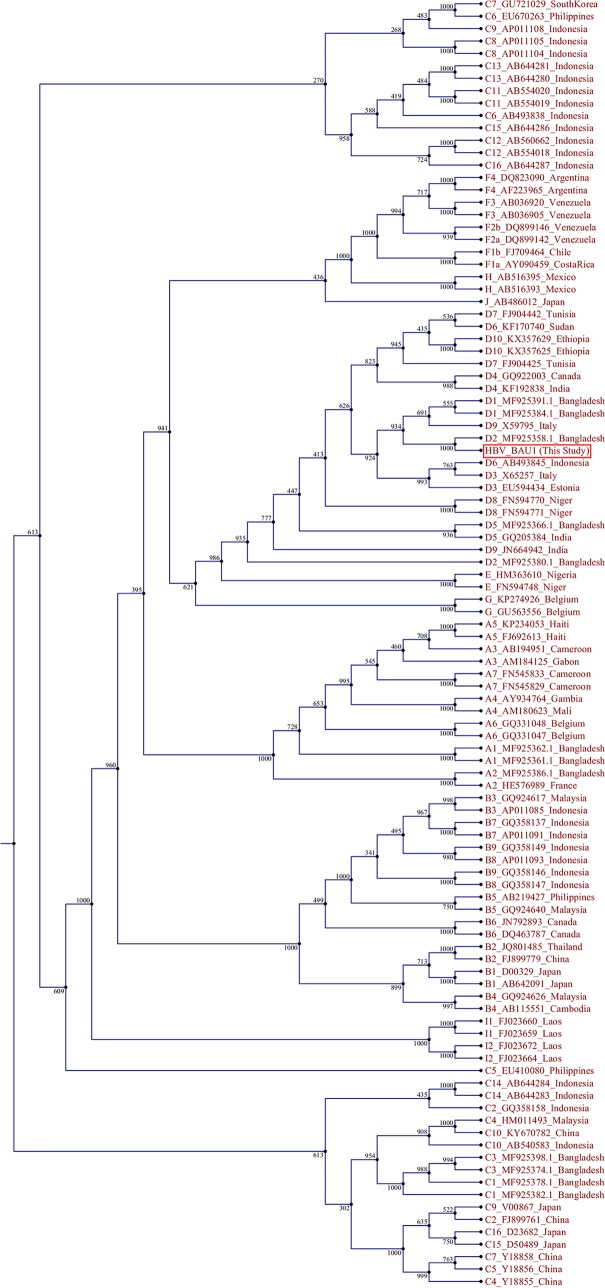
Phylogenetic tree analysis of HBV_BAU1 (GenBank accession no. MN839643). The full-genome sequences of different HBV strains were extracted from GenBank. The full-genome sequences were aligned and the phylogenetic tree was constructed using CLC Sequence Viewer version 6.8.1. The neighbor-joining algorithm was used with 1,000 replicates of bootstrap analysis to build the tree.

The entire assembled genome was circular and 3,182 bp long, with four overlapping ORFs, namely, P, S, C, and X. The minimum quality value (QV) scores of the reads used for the assembly were 20. The G+C content of the genome was 48.65%. This isolate showed a nonfunctional/defective pre-C protein due to a mutation at nucleotide position G1896A (insertion of stop codon TAG). The viral isolate was characterized as genotype D (subgenotype D2) and subtype (serotype) ayw3 and was evolutionarily related by phylogenetic analysis to the HBV isolated from Bangladesh, Italy, Indonesia, and Estonia (Fig. 1). A vaccine-escape HBsAg A128V mutation was found. The following nucleotide mutations were found: A2962G and C3026A in the pre-S1 region; T1753C, G1757A, and A1762C in the basic core promoter region; and G1896A and A2189T in the pre-C/C regions. The nonfunctional/defective pre-C protein of this isolate highlights the need for further investigation of this type of HBV in Bangladesh, along with its clinical outcome.

### Data availability.

The complete genome sequence of the isolated HBV strain HBV_BAU1 has been deposited in GenBank under the accession no. MN839643.
